# Smad3 is required for the survival of proliferative intermediate progenitor cells in the dentate gyrus of adult mice

**DOI:** 10.1186/1478-811X-11-93

**Published:** 2013-12-13

**Authors:** Silvia Tapia-González, Mª Dolores Muñoz, Mª Isabel Cuartero, Amelia Sánchez-Capelo

**Affiliations:** 1 CIBERNED – Ser. Neurobiología-Investigación, Hospital Universitario Ramón y Cajal-IRYCIS, Ctra. Colmenar Viejo Km 9, 28034 Madrid, Spain; 2 Unidad Neurología Experimental, Hospital Universitario Ramón y Cajal-IRYCIS, Ctra. Colmenar Viejo Km 9, 28034 Madrid, Spain

**Keywords:** Smad3, Adult neurogenesis, LTP, Hippocampus, Intermediate progenitor cell, Type 2 cells, Dentate gyrus, TGF-β

## Abstract

**Background:**

New neurons are continuously being generated in the adult hippocampus, a phenomenon that is regulated by external stimuli, such as learning, memory, exercise, environment or stress. However, the molecular mechanisms underlying neuron production and how they are integrated into existing circuits under such physiological conditions remain unclear. Indeed, the intracellular modulators that transduce the extracellular signals are not yet fully understood.

**Results:**

We show that Smad3, an intracellular molecule involved in the transforming growth factor (TGF)-β signaling cascade, is strongly expressed by granule cells in the dentate gyrus (DG) of adult mice, although the loss of Smad3 in null mutant mice does not affect their survival. Smad3 is also expressed by adult progenitor cells in the subgranular zone (SGZ) and more specifically, it is first expressed by Type 2 cells (intermediate progenitor cells). Its expression persists through the distinct cell stages towards that of the mature neuron. Interestingly, proliferative intermediate progenitor cells die in Smad3 deficiency, which is associated with a large decrease in the production of newborn neurons in Smad3 deficient mice. Smad3 signaling appears to influence adult neurogenesis fulfilling distinct roles in the rostral and mid-caudal regions of the DG. In rostral areas, Smad3 deficiency increases proliferation and promotes the cell cycle exit of undifferentiated progenitor cells. By contrast, Smad3 deficiency impairs the survival of newborn neurons in the mid-caudal region of the DG at early proliferative stages, activating apoptosis of intermediate progenitor cells. Furthermore, long-term potentiation (LTP) after high frequency stimulation (HFS) to the medial perforant path (MPP) was abolished in the DG of Smad3-deficient mice.

**Conclusions:**

These data show that endogenous Smad3 signaling is central to neurogenesis and LTP induction in the adult DG, these being two forms of hippocampal brain plasticity related to learning and memory that decline with aging and as a result of neurological disorders.

## Background

New neurons generated in the adult DG are constantly integrated into the hippocampal circuit. Several lines of evidences suggest that these newborn neurons are involved in learning and memory, particularly in pattern separation between similar contexts, a significant mechanism of memory formation [1-3], although it remains unclear how neurogenesis contributes to these cognitive processes. The rodent hippocampus displays a longitudinal (septotemporal) functional compartmentalization, whereby the rostral region is associated primarily with cognitive functions, and the more caudal regions with stress, emotion and affectivity [4]. This compartmentalization may produce different neurogenic environments that are associated with distinct rates of proliferation and/or differentiation [5,6]. In this sense, it appears that the increased survival of newborn neurons promoted by spatial learning tasks may be restricted to the rostral DG [7].

Adult neurogenesis is an active process, involving the proliferation of neural progenitors, cell fate specification, differentiation, maturation, migration and functional integration into the preexisting neuronal circuitry. In the adult DG, the cascade of neuronal differentiation is first characterized by the presence of a class of neural stem cells, radial glia-like (RGL) cells, believed to be largely quiescent and known to be nestin^+^GFAP^+^. Non-radial precursors represent another type of neural stem cells that are Sox2^+^GFAP^-^, that lack radial processes and that are more mitotic than RGLs, although most of them are not always in the cell cycle [8]. Asymmetric divisions of neural stem cells generate the amplifying of intermediate progenitor cells (or Type 2 cells), which exit the cell cycle within 1–3 days after several rounds of symmetric divisions to become post-mitotic neuroblasts (or Type 3 cells), which then differentiate into neurons [9,10].

The molecular mechanisms that govern these sequential developmental events in the adult DG are not completely understood, although the tight coordination between cell-intrinsic programs and external signals within the neurogenic niche seems to be required [11]. Indeed, extracellular signals that regulate survival and integration, such as the neurotrophic factors BDNF, FGF-2 and NT-3 [12-14], or the neurotransmitters GABA [12,15,16] and Glutamate [17,18], require intracellular modulators to transduce these signals. In this sense, it has been described the role of Prox1 in transducing Wnt singaling [19], CREB signaling in GABA-mediated excitation [20] or NFATc4 for BDNF-driven survival signaling [21] in adult hippocampal neurogenesis (AHN).

TGF-β1 is a pleiotropic cytokine highly expressed in neurodegenerative disorders like Parkinson’s or Alzheimer’s disease. We recently found that Smad3 deficiency, an intracellular molecule involved in TGF-β signaling cascade, promotes nigrostriatal dopaminergic neurodegeneration and α-synuclein aggregation [22]. Other studies have shown that the loss of TGF-β1 activity contributes to tau pathology and β-amyloid deposition [23], both pathologies associated with alterations in cognitive processes and AHN. Indeed, it has been suggested that dysfunctional neurogenesis may exacerbate neuronal vulnerability to the disease [24].

In this study we have addressed the role of Smad3 in adult DG neurogenesis and its impact on synaptic transmission. Previous studies in another neurogenic region, the subventricular zone, identified a reduction in proliferating cells in Smad3^ex8/ex8^ mice (a targeted deletion strategy to avoid activation of Smad3 by its receptor) and reduced migration to the olfactory bulb [25]. To study the DG, we have used a Smad3 null mouse in which there is a targeted deletion of the start codon and hence, no expression of the Smad3 protein [22]. In this model, we show that Smad3 deficiency promotes the death of intermediate progenitor cells. Furthermore, Smad3 provokes distinct effects on the rostral and middle-caudal regions of the DG. Accordingly, in the rostral domain there is enhanced proliferation and cell cycle exit of proliferative progenitor cells, which is not observed in the middle-caudal region. Furthermore, apoptosis is induced at intermediate progenitor cell stage, which strongly diminishes adult neurogenesis in the middle-caudal region. Indeed, Smad3 deficiency abolishes LTP formation in the DG, identifying Smad3 as a fundamental element driving cellular and synaptic plasticity in the DG.

## Results

### Smad3 deficiency does not alter granule neuron survival in the DG

**Figure 1:**
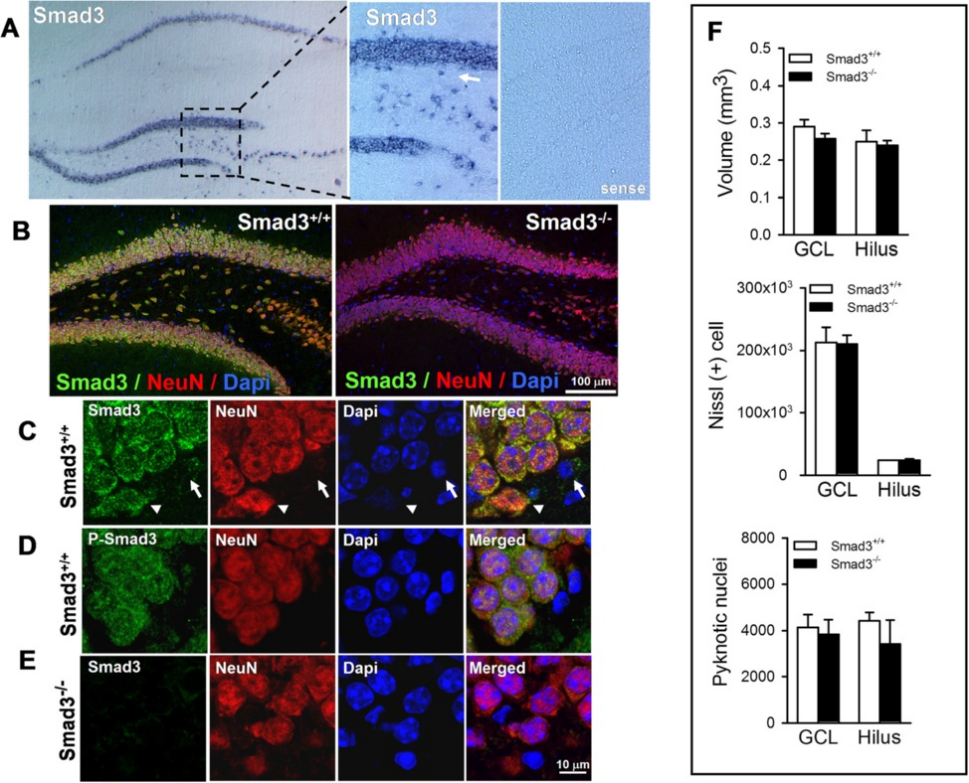
**Smad3 deficiency does not alter the survival of mature granule neurons in the DG. (A)** Smad3 mRNA expression was assessed by *in situ* hybridization on coronal sections of the adult hippocampus from wild-type mice. The arrow indicates Smad3 mRNA expression in cells located in the SGZ of the DG. The sense probe, used as a control, produced no staining. **(B)** Confocal images of Smad3/NeuN double labeling in the DG of Smad3^+/+^ and Smad3^-/-^ mice highlighting the generally normal morphology in Smad3 null mice. Scale bar 100 μm. **(C)** Smad3 is expressed by mature granule neurons of the DG, both in the cytoplasm and nucleus. In SGZ cells, strong (arrowhead) and weak (arrow) Smad3 expression was detected. **(D)** Phospho-Smad3 is present in the cytoplasm and nucleus of mature granule neurons. **(E)** Smad3 null mice do not express Smad3 protein. Sections from three mice were analyzed and representative images are shown. Scale bar 10 μm. **(F)** In the DG, unbiased stereological methods were used to estimate the volume and the number of Nissl stained neurons or that of pyknotic nuclei in Smad3^+/+^ and Smad3^-/-^ mice (n.s., student’s *t-*test, n = 6 mice per genotype).

The expression of the Smad3 transcription factor in the neurogenic region of the adult hippocampus has yet to be analyzed in detail. Through *in situ* hybridization using a specific probe against Smad3, we found Smad3 transcripts to be strongly expressed in the CA1-CA3, hilus and DG regions of the hippocampus. Indeed, cells expressing Smad3 were detected in the SGZ, the proliferative region of the DG (Figure 1A, arrow). The post-mitotic neuronal specific nuclear protein (NeuN) was co-expressed with Smad3 in the granular cells of the DG (Figure 1B). Indeed, the SGZ contained a mixed population of cells that expressed different levels of NeuN and Smad3 (Figure 1C, arrows), probably reflecting the process of neuronal maturation. Smad3 could be detected in both the cytoplasm and the nucleus of mature granule neurons. Indeed, phospho-Smad3 was also observed in these subcellular locations (Figure 1D), suggesting that the Smad3 signaling pathway may be active in these neurons.

As Smad3 null mice develop to adulthood [22], we took advantage of this genetic model to study the contribution of Smad3 to the adult DG. We previously showed that Smad3 promotes the postnatal survival of dopaminergic neurons in the substantia nigra [22]. Thus, to evaluate whether Smad3 imparts a survival signal to mature neurons of the granule cell layer (GCL), which are generated during embryonic development, we estimated the number of granule neurons in the DG of Smad3 knockout mice in the basal state. The hippocampus of Smad3^-/-^ mice had a generally normal morphology (Figure 1B, E), with no alteration in the volume of the DG (P = 0.220) or the hilus (P = 0.730) compared with Smad3^+/+^ littermates (Figure 1F). The number of Nissl stained neurons estimated by unbiased stereological methods was similar in Smad3^+/+^ and Smad3^-/-^ mice (P = 0.941). Furthermore, a similar number of pyknotic nuclei were evident in the GCL (P = 0.734) and the hilus (P = 0.398) of both genotypes, suggesting that cell death was not altered by the Smad3 deficiency. These results suggest that the prosurvival effect of Smad3 in dopaminergic neurons of the adult substantia nigra was not observed in other regions, such as the hippocampal GCL or striatum. Furthermore, Smad3 does not seem to play a central role during the development of these three brain regions [22].

### Smad3 is expressed in SGZ progenitors

**Figure 2:**
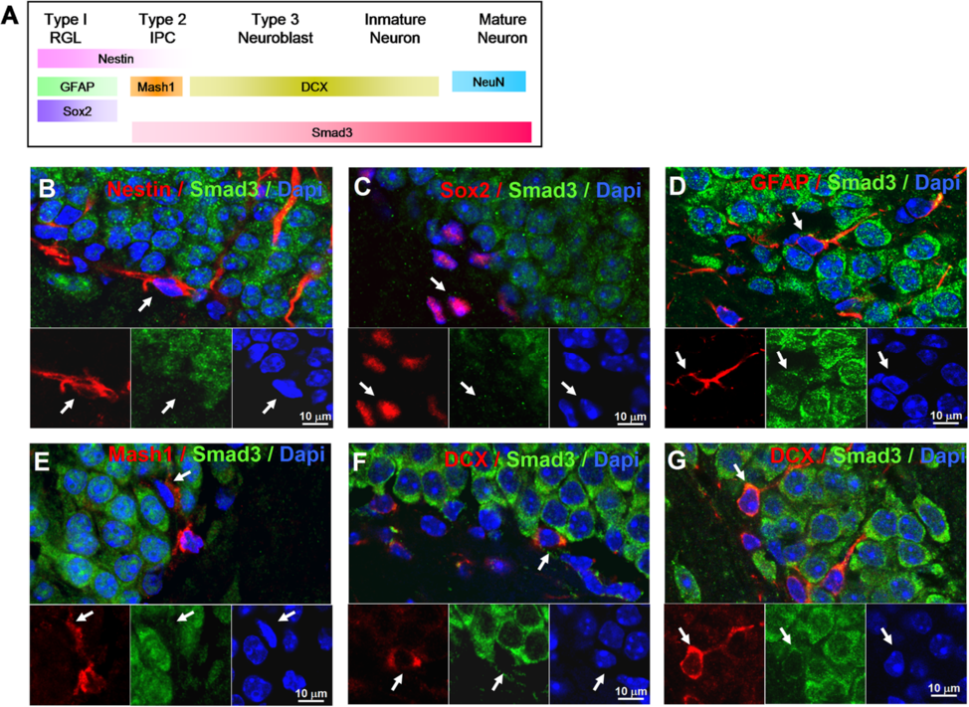
**Expression of Smad3 at different stages of neuronal precursor maturation. (A)** In the prevailing model of AHN, quiescent radial glia-like cells (RGL or Type-1 cells) generate proliferative precursors known as intermediate progenitors cells (Type-2 cells), which give rise to neuroblasts (Type-3 cells), then to immature neurons and finally generating mature granule neurons. The identification of the different type of cells is based on the expression of specific precursors and lineage markers. Confocal microscopy images of **(B)** Nestin/Smad3, **(C)** Sox2/Smad3, **(D)** GFAP/Smad3, **(E)** Mash1/Smad3, **(F)** DCX/Smad3 double-labeled cells with no dendrite maturation, indicative of Type 2b or neuroblast cells, and **(G)** with dendrite maturation indicative of immature neurons. Scale bar 10 μm.

We evaluated whether the expression of Smad3 in the SGZ (Figure 1A, arrow) might be related to the neurogenic processes in this region. By studying the immunolabeling for different markers and using confocal microscopy, we assessed the expression of Smad3 at specific stages of neuronal maturation: in quiescent RGL (Type-1) cells, intermediate progenitor cells (Type-2 cells), neuroblasts (Type-3 cells), immature neurons, and in mature granule neurons (Figure 2A) [10]. Although we could detect Smad3 expression in GFAP(+) cells with a morphological extension resembling a radial branch (Figure 2D), no Smad3 expression could be observed in nestin(+) or Sox2(+) cells (Figure 2B-C). Similar results were found using the anti-phospho-Smad3 antibody (data not shown), suggesting Smad3 was not expressed in either RGL or non-radial neural precursors. However, we could detect weak expression of Smad3 in Mash1(+) cells, which are early intermediate progenitor cells (Figure 2E). Indeed, Smad3 was detected in cells labeled for doublecortin (DCX) with different morphologies, from DCX(+) cells with a rounded or flattened nuclear morphology (Figure 2F), possibly representing late phases of type-2 cells and neuroblast stages, to DCX(+) cells with clear dendrite maturation that may represent immature neurons (Figure 2G) [26,27]. Indeed mature neurons labeled with NeuN (Figure 1B-D) also expressed Smad3, suggesting that Smad3 is expressed at the neuroblast, immature and mature granule neuron stages.

These data suggest that Smad3 is not present in RGL or non-radial neural precursors but rather, that it begins to be expressed by intermediate progenitor cells, and that it persists through to the stage of the mature neuron.

### Rostral increase in proliferative cells in the absence of Smad3

**Figure 3:**
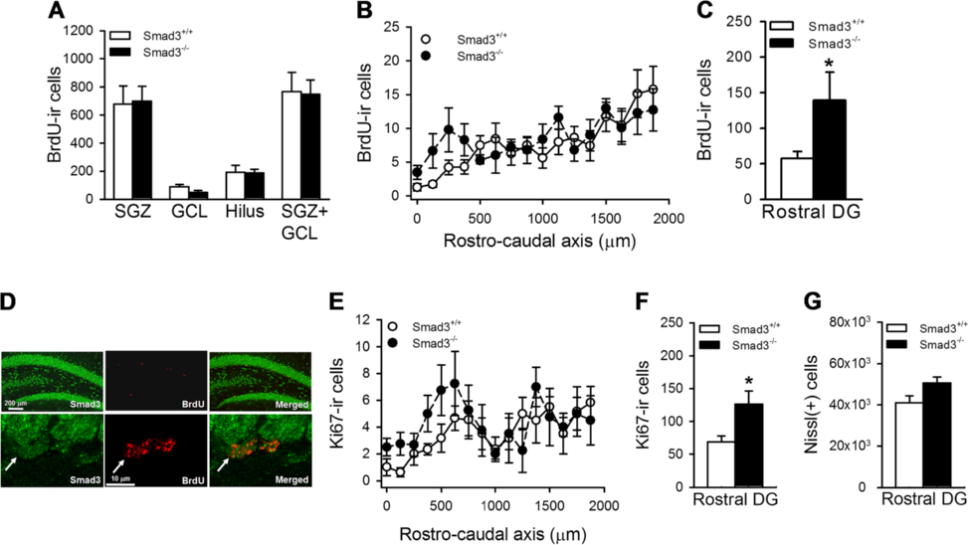
**Smad3 promotes the proliferation of NPCs in the rostral DG.** To analyze the proliferation of progenitor cells, mice received a daily injection of BrdU (100 mg/Kg) over 5 consecutive days and they were sacrificed 2 days after the final injection. **(A)** Smad3 deficiency did not affect the number of BrdU-ir cells in the SGZ, GCL or the hilus. **(B-C)** Representation of the SGZ along the rostro-caudal axis showed that Smad3^-/-^ mice had more BrdU-ir cells in the initial rostral 500 μm but not in the middle and caudal regions of the SGZ (*, P ≤ 0.05, Student *t*-test, n = 5-7 mice per genotype). **(D)** Confocal images showing Smad3 expression in BrdU labeled proliferative cells of the SGZ. Scale bar 200 μm and 10 μm. **(E-F)** The endogenous marker of cell proliferation Ki-67 further confirmed the enhanced progenitor cell proliferation in the initial 750 μm rostral portion of the DG in Smad3^-/-^ mice compared to that in Smad3^+/+^ mice (*, P ≤ 0.05, Student’s *t*-test, n = 5-6 mice per genotype). **(G)** The number of Nissl stained neurons in the rostral DG showed a non-statistical trend to increase in Smad3^-/-^ mice in the rostral DG (P = 0.054, Student’s *t*-test, n = 6 mice per genotype).

**Figure 4:**
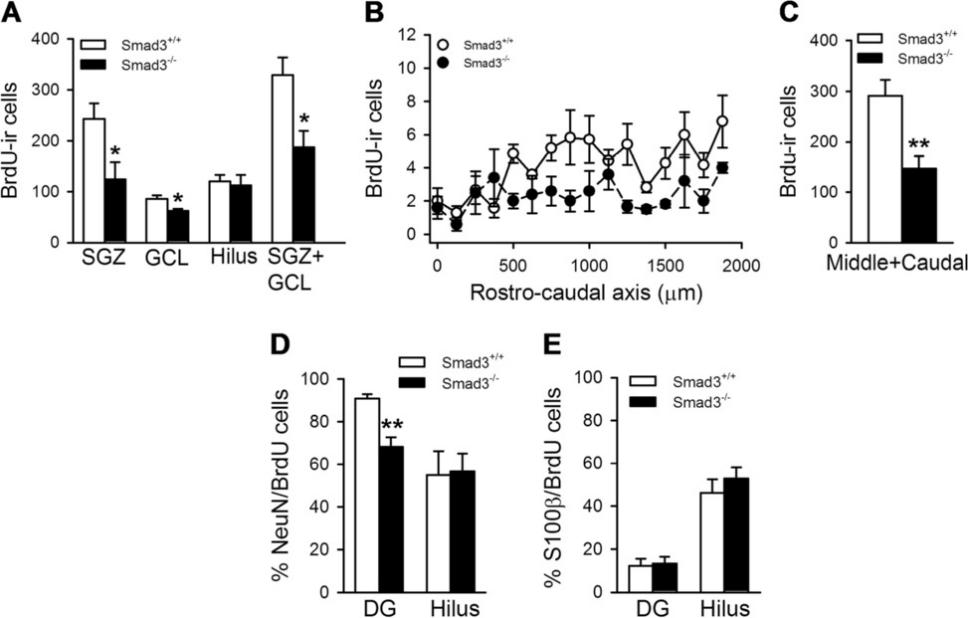
**Smad3 deficiency decreases adult neurogenesis in the DG.** Mice received a daily injection of BrdU (100 mg/Kg) on five consecutive days and the survival of newborn cells was evaluated 28 days after the last injection. **(A)** Effect of Smad3 deficiency on the survival of newborn cells labeled with BrdU in the SGZ, GCL and the hilus. **(B-C)** Representation of the DG (SGZ + GCL) along the rostro-caudal axis showed that Smad3^-/-^ mice had fewer BrdU-ir cells than Smad3^+/+^ in the middle and caudal regions, but not in the rostral regions (*, P ≤ 0.05; **, P ≤ 0.01 Student’s *t*-test, n = 5-7 mice per genotype). **(D)** Effect of Smad3 deficiency on progenitor differentiation to neurons (BrdU/NeuN) and **(E)** astrocytes (BrdU/S100β; **, P ≤ 0.01, Student’s *t*-test, n = 4-5 mice per genotype).

Given the presence of Smad3-immunoreactive (-ir) precursor cells in the adult SGZ, we hypothesized that this signaling pathway could mediate an intrinsic mechanism that regulates AHN. We examined Smad3 expression in proliferating cells identified by *in vivo* BrdU labeling of dividing cells, and we found Smad3 to be expressed in BrdU-ir cells in the SGZ, GCL and the hilus of mice (Figure 3D). To determine whether Smad3 might influence cell proliferation in the DG, mice received five daily BrdU injections and they were then sacrificed 2 days after the last injection. We estimated the number of BrdU-labeled cells and we found no overall difference in the number of proliferative precursor cells in the SGZ, GCL or hilus (Figure 3A), nor when we considered both regions of the DG (SGZ + GCL) of Smad3-deficient and wild-type mice (Smad3^+/+^, 709.5 ± 105.9; Smad3^-/-^, 739.3 ± 78.87; P = 1.000). However, when these values were expressed along the rostrocaudal axis of the SGZ, we observed a 2.42-fold increase in BrdU-ir cells in the rostral portion of Smad3^-/-^ mice with respect to those in wild-type mice (first 500 μm; Smad3^+/+^, 57.7 ± 9.8; Smad3^-/-^, 139.3 ± 39.6; P = 0.041; Figure 3B-C). To confirm this, we examined the endogenous marker of proliferation Ki-67. While there was also a similar total number of cells expressing Ki-67 in the DG of Smad3^-/-^ mice and their Smad3^+/+^ littermates (Smad3^+/+^, 301.0 ± 53.0; Smad3^-/-^, 336.3 ± 21.6; P = 0.594), the rostral portion of the DG had 83% more Ki-67-ir cells in Smad3^-/-^ mice than in Smad3^+/+^ mice (first 750 μm; Smad3^+/+^, 69.0 ± 9.1; Smad3^-/-^, 126.3 ± 20.5; P = 0.020; Figure 3E-F). We re-examined the number of Nissl stained cells in this portion of the DG to search for a rostral increase in the number of mature granule neurons. We detected a trend towards an increase in the number of granule neurons in Smad3 deficient mice (23.8%) compared with their control littermates (first 500 μm; Smad3^+/+^, 40986 ± 3406; Smad3^-/-^, 50797 ± 2823; P = 0.059; Figure 4F), although this strong trend did not quite reach statistical significance. Overall, these results suggest that although Smad3 is expressed in progenitor cells along the rostrocaudal axis of the DG, it inhibits proliferation in the rostral but not in the middle or caudal regions of the DG.

### Smad3 is critical for adult neurogenesis in the DG

The survival of the progeny of progenitor cells was examined 28 days after the last BrdU injection. To define the survival ratio, we compared the number of BrdU-ir cells observed at 28 days to those counted 2 days after BrdU injection. Smad3^+/+^ mice had 57.1% fewer BrdU-ir cells in the DG after 28 days (Figure 4A; 329.0 ± 34.5; P < 0.001) than on day 2 after injection (Figure 3A). However, in Smad3^-/-^ mice there were 75.1% fewer BrdU-ir cells on day 28 (186.2 ± 32.9; p < 0.001), representing a 43.3% reduction in their survival (P = 0.016). There was no difference in the cell diameter of granule neurons between these mice (Smad3^+/+^, 7.84 ± 0.23 μm; Smad3^-/-^, 8.29 ± 0.21 μm; P = 0.145). In the hilus no significant differences in BrdU-ir cell number were detected between the groups. The poorer survival of BrdU-ir cells in Smad3^-/-^ mice was evident in the middle and caudal regions of the DG (Figure 4B-C; Smad3^+/+^, 291.5 ± 31.3; Smad3^-/-^, 146.9 ± 24.0; P = 0.007), while similar survival was detected in the rostral portion of the null mutants and their wild-type littermates (first 500 μm; Smad3^+/+^, 37.6 ± 8.9; Smad3^-/-^, 40.5 ± 12.9; P = 0.930). The distribution of newborn cells in the SGZ and GCL was not obviously different between the two genotypes and thus, both regions of the DG were considered in the rest of the analyses.

We investigated the differentiation fate of the precursor cells by double-labeling with BrdU and with neuronal (NeuN) or astrocyte (S100β) markers. The percentage of progenitor cells that differentiated into astrocytes did not differ between Smad3^+/+^ (12.2 ± 3.4%) and Smad3^-/-^ mice (13.2 ± 3.3%; P = 0.850; Figure 4E). By contrast, the percentage of BrdU-ir cells that were also labeled for NeuN was 68.3 ± 4.34% in Smad3^-/-^ mice, significantly lower than in wild-type mice (90.8 ± 2.1%, P = 0.009). Overall, the number of neurons produced in the Smad3^-/-^ mice in one month was 44.4% less than in their wild-type littermates (Smad3^+/+^, 377.8 ± 15.0; Smad3^-/-^, 210.0 ± 37.1; P = 0.014), suggesting a key role for Smad3 intracellular signaling in AHN.

### Cell cycle progression

**Figure 5:**
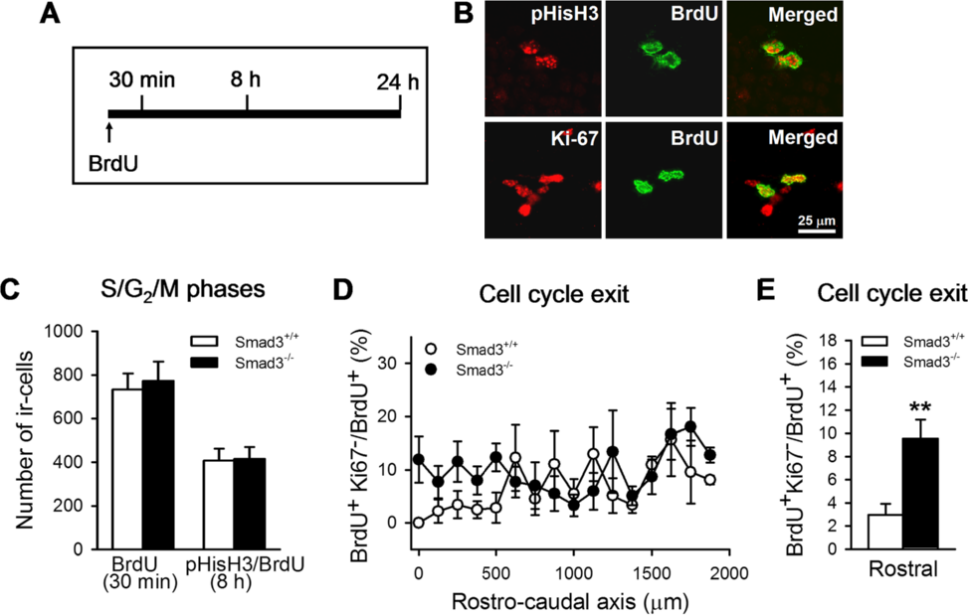
**Cell cycle progression in Smad3**^**-/- **^**mice. (A)** Scheme of the experimental design to study cell cycle progression in Smad3 deficient mice. Mice were sacrificed 30 minutes, 8 hours and 24 hours after receiving a single dose of BrdU (150 mg/Kg). **(B)** Confocal images of BrdU and pHisH_3_ double-labeled cells 8 hours after pulse-labeling with BrdU. BrdU/Ki-67 double-labeled cells were assessed 24 hours after BrdU injection. Scale bar 25 μm. **(C)** Number of cells in S phase (labeled with BrdU) 30 minutes after delivering the BrdU pulse, and of cells in the G_2_/M phase (double-labeled with BrdU and pHisH_3_) 8 hours after pulse labeling (n.s., two-way ANOVA n = 3-5 mice per genotype). **(D-E)** The index of cell cycle exit in wild-type and Smad3^-/-^ mice showing an increase in the rostral DG. The index of cell cycle exit was scored by defining the ratio between BrdU^+^Ki67^-^ cells and total BrdU^+^ cells in the DG, which corresponds to the fraction of precursors that have left the cell cycle within 24 hours (**, P ≤ 0.01, Student’s *t*-test, n = 5 mice per genotype).

It is well established that TGF-β signaling has cytostatic activities and in particular, that Smad3 inhibits cell cycle progression from the G_1_ to S phase [28,29]. Hence, we sought to determine whether the decrease in neurogenesis associated with Smad3 deficiency was related to alterations in the cell cycle progression of progenitor cells. We examined whether the proportion of progenitor cells in the S phase or G_2_/M phases of the cell cycle, and their index of cell cycle exit, was altered in Smad3 null mice. The mitotic activity of progenitor cells was evaluated through the incorporation of BrdU in pulse-labeling assays. Wild-type and Smad3 null mice were analyzed 30 minutes after a single BrdU injection (150 mg/Kg, Figure 5A), a labeling regime that is sufficient to saturate the proliferative cells in S phase. A second group of mice was analyzed 8 hours after the same dose of BrdU was administered, double-labeling BrdU-ir cells with pHisH_3_, a marker of the G_2_/M phases of the cell cycle. At this time point after the BrdU pulse, the co-localization of BrdU and pHisH_3_ is maximal [30,31]. Finally, mice were analyzed 24 h after BrdU labeling to study cell cycle exit, through the co-localization of BrdU and Ki-67, a marker of the G_1_/S/G_2_/M phases that is downregulated after cell cycle exit [32].

The number of BrdU-ir cells detected in the DG 30 minutes after pulse-labeling was similar in Smad3^-/-^ mice and their control littermates (Smad3^+/+^, 734.2 ± 76.8; Smad3^-/-^, 773.8 ± 128.8; Figure 5C), as were the number of precursor cells that had entered the G_2_/M phases 8 hours after the BrdU pulse. Accordingly, there were 407.5 ± 28.8 and 415.5 ± 65.1 BrdU^+^pHisH3^+^ cells in Smad3^+/+^ and Smad3^-/-^ mice, respectively [two-way ANOVA, *F*_(1, 14)_ = 0.001; no effect of genotype (P = 0.822), time after BrdU labeling (P = 0.118) or interaction (P = 0.972)]. These results suggest that in progenitor cells, the S/G_2_/M phases of the cell cycle were not altered in Smad3 deficient mice.

In standard situations, cells divide as they pass through the M phase of the cycle, while some exit the cell cycle at G_1_ and others re-enter in S phase. We scored the index of cell cycle exit 24 hours after pulse-labeling with BrdU, defining the ratio between BrdU^+^Ki67^-^ cells and the total numbers of BrdU^+^ cells in the DG, which corresponds to the fraction of precursors that have left the cell cycle within 24 hours [32-34]. There was a significant increase in the cell cycle exit index in the rostral portion of Smad3^-/-^ relative to control mice (Smad3^+/+^, 2.98.40 ± 0.94%; Smad3^-/-^, 9.55 ± 1.63%; P = 0.008) but not in the middle and caudal regions (Figure 5D-E), confirming the different behavior of progenitor cells in these areas of the DG. Together these data suggest that Smad3 deficiency does not alter cell cycle progression through S/G_2_/M, although inactivating Smad3 signaling in the rostral DG affects the decision to exit the cell cycle.

### Fewer intermediate progenitor cells in Smad3 deficient mice

**Figure 6:**
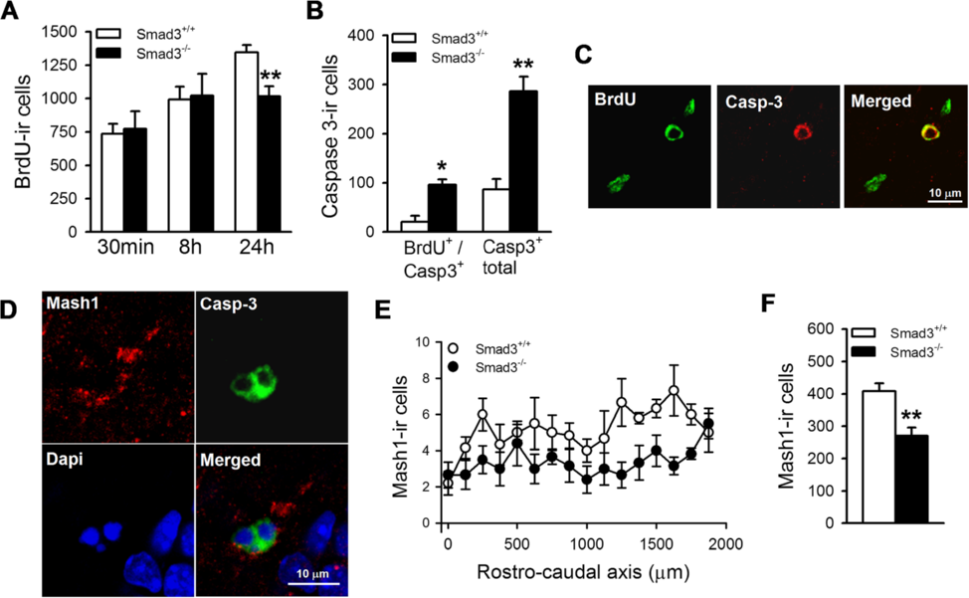
**Smad3 deficiency leads to apoptotic cell death and fewer intermediate progenitor cells. (A)** The total number of BrdU-ir cells 30 minutes, 8 hours and 24 hours after delivering the BrdU pulse (150 mg/Kg: **, P ≤ 0.01, two-way ANOVA, Holm-Sidak *post hoc* test n = 3-5 mice per genotype). **(B)** Histograms showing the total number of activated caspase 3-ir cells and those double-labeled with BrdU in the DG (*, P ≤ 0.05, **, P ≤ 0.01, Student *t*-test, n = 3 mice per genotype). **(C)** Confocal images of activated caspase 3/BrdU double-labeling, 24 hours after BrdU injection showing apoptotic BrdU-ir cells in the DG. Scale bar 10 μm. **(D)** Confocal image showing nuclear activated caspase 3 in intermediate progenitor cells expressing Mash1 in the cytoplasm. Cell shrinkage morphology, nuclear condensation and fragmentation and activated caspase 3 are suggestive of apoptosis. Scale bar 10 μm. **(E-F)** Quantification of the number of Mash1(+) cells in the DG along the rostro-caudal axis showed that Smad3^+/+^ mice had fewer intermediate progenitor cells (**, P ≤ 0.01, Student *t*-test, n = 6 per genotype).

We next focused on the total number of BrdU-ir cells present 30 min, 8 h and 24 h after pulse labeling mice (Figure 6A). Two-way ANOVA analyses showed an interaction between genotype and time after BrdU pulse labeling (*F*_(2, 23)_ = 3.936; P = 0.038). There was no difference between Smad3^+/+^ and Smad3^-/-^ in terms of the total number of BrdU-ir cells present 30 minutes (P = 0.843) or 8 hours (P = 0.834) after pulse labeling. However, there were 24.5% fewer BrdU-ir cells 24 hours after pulse labeling in Smad3^-/-^ mice than in their Smad3^+/+^ littermates (Smad3^+/+^, 1345.8 ± 54.45; Smad3^-/-^, 1016.4 ± 75.9; P = 0.004). To evaluate whether progenitor cells might undergo apoptosis in the absence of Smad3 signaling at this early stage, we analyzed the expression of activated caspase 3 in the DG. The number of apoptotic cells increased significantly in Smad3^-/-^ mice compared to their wild-type littermates, as evident through both the total number of cells expressing activated caspase 3 (Smad3^+/+^, 100.0 ± 19.1; Smad3^-/-^, 371.7 ± 42.6; P = 0.004) and through the number of apoptotic BrdU-ir cells (Smad3^+/+^, 20.0 ± 12.6; Smad3^-/-^, 95.8 ± 11.2; P = 0.011; Figure 6B-C). Thus, the net reduction in the production of newborn adult hippocampal neurons observed in Smad3-deficient mice (Figure 4) seems to be the result of activating the apoptotic program in undifferentiated progenitor cells.

Because Smad3 is first expressed at the intermediate progenitor cell stage, we examined whether these proliferative cells might be dying in the absence of Smad3. Indeed, we could detect active caspase 3 in Mash1(+) cells (Figure 6D) and Smad3^-/-^ mice had 33.9% fewer Mash1(+) cells in the DG than Smad3^+/+^ littermate mice (Smad3^+/+^, 407.9 ± 25.0; Smad3^-/-^, 269.8 ± 26.3; P = 0.003; Figure 6E-F). Together, these data indicate that Smad3 signaling is central to AHN, regulating the survival of proliferative intermediate progenitor cells along the rostro-caudal axis.

### LTP is abolished in Smad3-deficient mice in the DG

**Figure 7:**
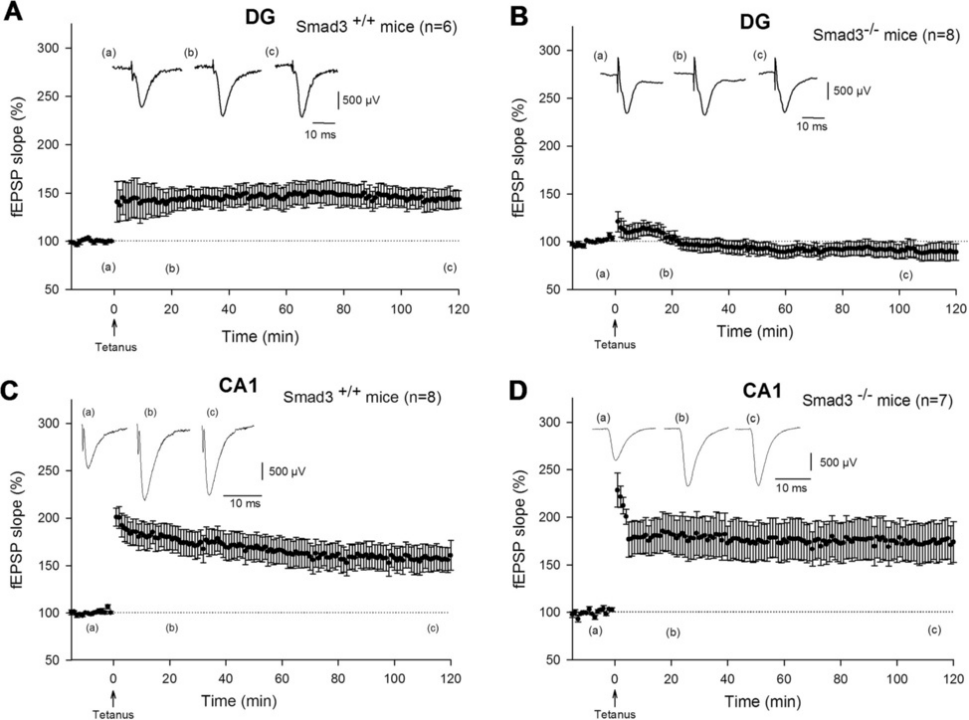
**Smad3 deficiency abolishes LTP induction in the DG after HFS of the MPP. (A)** LTP induction in the DG of Smad3^+/+^ mice. **(B)** Smad3^-/-^ mice do not exhibit evoked LTP in the DG. **(C-D)** HFS induces LTP in the CA1 region of Smad3^+/+^ and Smad3^-/-^ mice. Raw fEPSP data (a) before tetanus, (b) 15 min and (c) 120 min after tetanus administration.

We evaluated the impact of the decreased neurogenesis provoked by Smad3 deficiency on hippocampal LTP. The strongest induction of LTP in the DG was obtained by applying HFS to the MPP [35], which in wild-type mice resulted in a rapid and stable potentiation of the evoked field excitatory postsynaptic potentials (fEPSPs) slope (146.0 ± 11.1%, P < 0.001; Figure 7A). In Smad3 knockout mice, HFS was not followed by induction of LTP (94.0 ± 7.2%, P < 0.460; Figure 7B). In contrast to the DG, HFS of the Schaffer collateral pathway evoked LTP in the stratum radiatum of the CA1, both in Smad3^+/+^ mice (164.8 ± 13.8%, P < 0.001; Figure 7C) and in Smad3^-/-^ mice (176.2 ± 20.6%, P < 0.001; Figure 7D). Control experiments showed that these effects were not caused by electrode-induced injury, synaptic activation or granule cell DG discharges, because there was no significant difference in the input/output curves for the same group of animals (data not shown).

## Discussion

For the first time, we have identified a central role for Smad3 signaling in controlling the survival of proliferative intermediate progenitor cells and the rate at which newborn neurons are produced in the adult DG. Notably, Smad3 is not required for the survival of mature granule neurons generated during embryonic development, as also observed for neurons in other brain regions [22]. Moreover, Smad3 deficiency abolishes LTP in the DG, while LTP induction in the CA1 is evoked correctly, indicating a central role for Smad3 in DG cellular and synaptic plasticity.

Smad3 is not present in neural stem cells (radial and non-radial precursors) labeled with Sox2 or nestin markers. We have found a small number of GFAP^+^Smad3^+^ cells with a cell body residing in the DG and a radial process extending into the granule layer, however they seems not to be RGL precursors. It is already known the heterogeneity of RGL cells, with subpopulations identified by different markers [36] or properties, such as proliferative or quiescence, although with similar morphology [10]. However, we have found GFAP^+^Smad3^+^ cells near vessels, suggesting that they could be astrocytes. Further studies will clarify whether these cells might meet the specific criteria for stem cells or could be classified as astrocytes. Smad3 co-localizes with Mash1, a specific marker of intermediate progenitor cells, and its expression persists in neuroblasts, immature and mature neurons. Intermediate progenitor cells are the most proliferative cell type in the adult DG [10], and this expression suggest that Smad3 may have a distinct effect on the proliferative capacity of intermediate progenitor cells and post-mitotic newborn neurons. Indeed, proliferative intermediate progenitor cells die in the absence of Smad3, inducing a strong decrease in the rate at which newborn neurons are generated.

Through BrdU pulse-labeling we found that the S and G_2_/M phases of the cell cycle are not modified in the absence of Smad3. However, inactivating Smad3 signaling in the rostral DG increases the decision to exit the cell cycle, as detected 24 h after BrdU pulse-labeling through the co-localization of BrdU and Ki-67. Ki67 is a marker of proliferation expressed during the G_1_/S/G_2_/M phases of the cell cycle, which it is downregulated after cell cycle exit and absent in resting cells [32-34]. The short half-life of Ki67, estimated to be around 60 to 90 minutes [37,38], avoids the accumulation of non-degraded protein soon after cell cycle exit. Considering that the cell cycle length of precursor cells in the DG is 14 hours [31], Ki67 labeling of cells that incorporated BrdU 24 hour previously allows progenitor cells that have left the cell cycle to be discerned. The observed result suggests a potential role for Smad3 in regulating G_1_ phase, where the cell cycle exit decision is made [39]. BrdU pulse-labeling also shows that Smad3 deficient cells die through apoptosis 24 h after injection. It is already known that the majority of proliferating cells that have incorporated BrdU 2 to 24 hours after injection are intermediate progenitor cells [40,41], and it is estimated that intermediate progenitor cells can pass through up to five cell cycles as transient amplifying cells [42]. Hence, we might envisage that transit amplifying intermediate progenitor cells deficient in Smad3 signaling activate apoptosis during their cell cycle.

TGF-β/Smad3 signaling is known to fulfill a central role in cell cycle progression and apoptosis [43]. TGF-β signaling inhibits cell growth through its cytostatic activity. In particular, Smad3 inhibits the progression of epithelial cells from the G_1_ to S phase of the cell cycle, and in other cell types albeit to a lesser extent [29]. Smad3 induces cell cycle arrest by inhibiting cyclin-dependent kinases (Cdk) and key transcriptional regulators, such as c-Myc and the Id family of proteins [28]. In the early G_1_ phase, cell cycle progression is driven by the concerted action of Cdk4 and Cdk6, whereas Cdk2 is the driving force during late G_1_ phase [44]. It has already been shown that Cdk6, but not Cdk4 or Cdk2, is required for AHN [33,45]. In addition, to ensuring correct progression through the cell cycle, there are internal checkpoints that monitor the conditions to generate healthy daughter cells, and cell damage or stress may restrict cell cycle progression and/or induce cell death, through them. The G_1_/S checkpoint protects genomic integrity and prevents damaged cells from entering S-phase by inducing apoptosis [46-48]. In this sense, alteration to the G_1_/S checkpoint in the absence of Smad3 could lead intermediate progenitor cells to undergo apoptosis. Thus, one might envisage a model where Smad3 is actively involved in ensuring that proliferative precursors pass the G_1_/S checkpoint, preventing them from undergoing premature apoptosis and diminishing neurogenesis. Considering that the transition from intermediate progenitor cells to neuroblasts is the main critical period in the survival of newborn cells [49], Smad3 signaling appears to be a key molecule for the survival of newborn neurons.

It is notable that Smad3 deficiency produces different effects on progenitor cells in the rostral or middle-caudal regions of the DG. In the rostral portion, the increase in proliferation, and in cell cycle exit, induced by Smad3 deficiency was not translated into a clear increase in newborn neurons, probably due to the activation of apoptosis and cell death at the intermediate progenitor cell stage. Since newborn neurons contributes to only a minor fraction of the adult DG granule cell population [50,51], this would explain why no clear increment in the number of mature granule neurons can be observed in the rostral DG. On the other hand, the rostral increase in proliferation could be a mechanism to compensate for cell death and to maintain cell numbers in the rostral DG. Conversely, increased proliferation is not observed in the middle-caudal region and the induction of apoptosis at the intermediate progenitor cell stage strongly dampens adult neurogenesis.

An interaction of Smad3 with rostral or middle-caudal specific signals may account for this differential effect. On the other hand, a gradient of Smad3 expression may exist. Indeed, during development Smad3 regulates axis formation through Nodal, one of the best characterized morphogens involved in anterior-posterior embryonic patterning [52]. However, we could not detect stronger Smad3 expression in the rostral portion of the DG of adult mice (data not shown), nor in other areas of the brain such as the substantia nigra, a structure where we previously detected a rostral effect of Smad3 on the survival of dopaminergic neurons [22], suggesting a functional spatial differentiation of this signaling molecule. The longitudinal functional compartmentalization of the hippocampus may produce different neurogenic environments, in which distinct rates of proliferation and/or differentiation may be established [5-7]. The different effect of Smad3 on adult neurogenesis along the rostrocaudal axis of the DG might participate in this functional longitudinal compartmentalization of the hippocampus, promoting a distinct neurogenic environment in rostral regions.

Several ligands may activate Smad3, such as TGF-β1, -β2, -β3, activin or GDF1. It is already known that through Smad2/3 signaling, TGF-β controls self-renewal and differentiation in several types of stem cells [53]. Indeed, the role of TGF-β in AHN has been investigated using exogenous administration or transgenic overexpression of this cytokine, although with contradictory results that might depend on the experimental model [54-56]. Bearing in mind that the effects of TGF-β1 are dose- and context-dependent [43], its overexpression may introduce a bias in studies carried out under physiological conditions. Activin overexpression has also produced conflicting results when studying adult DG neurogenesis. In activin transgenic mice there is apparently no effect on neurogenesis [57], although i.c.v. infusion of the protein led to an increase in precursor cell number in another study [58]. When activin is experimentally inhibited by using its high-affinity antagonist follistatin, a marked decrease in neurogenesis is observed in uninjured transgenic mice and following i.c.v. infusion after excitotoxic neurodegeneration. However, follistatin may also have antagonist activity against other members of the TGF-β superfamily, such as TGF-β1, BMP4 or BMP7 [59,60], raising the possibility that the inhibitory effect of follistatin on neurogenesis could be mediated by other TGF-β molecules. In this sense, the extracellular ligand that may activate Smad3 in the DG to inhibit adult neurogenesis remains unknown.

Smad3 deficiency abolishes LTP induction in the DG and the specificity of this effect is evident as LTP is evoked properly in the CA1 region. A role for TGF-β in neuronal plasticity has been witnessed in *Drosophila* and *Aplysia*[61,62]. In the rodent hippocampus, Smad4 - which binds to diverse intracellular Smads that are involved in both TGF-β (Smad2 and Smad3) and BMP (Smad1, Smad5 and Smad8) signaling - does not participate in the induction of LTP in the CA1 hippocampal formation, although it does influence excitatory/inhibitory transmission, an effect that seems to be related to BMP signaling [63]. The effect of Smad3 deficiency on LTP that we detected could involve the NMDA or GABA receptor, the latter being implicated in neurogenesis. In the adult mouse brain, GABAergic transmission regulates proliferation, and it promotes the differentiation, maturation and functional integration of newborn neurons into the DG. Indeed, GABAergic input promotes the differentiation of amplifying intermediate progenitor cells [16,64,65]. Learning and memory formation rely on the experience-related modification of synaptic structures in the hippocampus and on the induction of LTP, an activity-dependent change in the synaptic strength [66]. It has already been shown that the *in vivo* induction of LTP at MPP inputs to the DG promotes neurogenesis [67], and conditional ablation of adult neurogenesis impairs LTP at MPP synapses [68], suggesting that these two processes are functionally linked. Whether the effect of Smad3 on neurogenesis might represent a link between these processes or if they are regulated in an independent manner will require further study. Furthermore, although Smad3 deficiency does not alter the number of mature granule neurons generated during embryonic development, we could not exclude a developmental alteration, and that glial cells deficient in Smad3 could also influence the observed effects. However, Smad3 seems to be a major contributor to both neurogenesis and LTP induction in the adult DG, these being two forms of hippocampal brain plasticity related to learning and memory.

Physiological conditions such as external aversive or enriching experiences, including stress or learning, may influence both neurogenesis and LTP [69,70]. Cycling intermediate progenitor cells have been seen to be the target of neurogenic external stimuli, such as running [40,41]. In this sense, Smad3 may participate in the physiological events regulated by neurogenesis. Indeed activin modulates anxiety and depression responses in mice [71,72]. On the other hand, different brain pathologies alter AHN, such as epilepsy, stroke, inflammation or neurodegeneration [73], and different forms of dementia and alterations in hippocampal neurogenesis are associated with Parkinson’s and Alzheimer’s disease [24]. Smad3 deficient mice represent an interesting model to study parkinsonism due to the effects on nigrostriatal dopaminergic neurodegeneration and α-synuclein aggregation [22], where overexpression of α-synuclein may also play a role on hippocampal neurogenesis [74]. Further studies will clarify whether this effect of Smad3 on hippocampal LTP and neurogenesis may be related to pathological events.

## Conclusions

We show here for the first time that endogenous Smad3 signaling is a major contributor to neurogenesis and LTP in the adult DG, highlighting its role in the intrinsic mechanisms that govern neuronal precursors and hippocampal plasticity.

## Methods

### RNA *In situ* hybridization

All steps for RNA *in situ* hybridization were performed in a RNase free environment, as described by Young and Mezey [75]. To generate DIG-labeled antisense and sense riboprobes, a cDNA fragment of Smad3 (GenBank accession number NM_016769) was generated by PCR and cloned into the XhoI/BamHI sites of pCRII (Invitrogen, Carlsbad, California, USA). The linearized plasmid served as the template for *in vitro* transcription using the DIG RNA Labeling Kit and the SP6/T7 RNA polymerases (Roche Diagnostics GmbH, Basel, Switzerland). The sense probe was used as a control and produced no staining.

Unfixed fresh-frozen brains were cryoprotected in 30% sucrose in PBS and embedded in optimal cutting temperature (OCT) compound (Sakura Finetechnical, Tokyo, Japan). Coronal cryostat sections (12 μm) were collected on microscope slides and they were post-fixed in 4% paraformaldehyde, permeabilized with proteinase K, acetylated, dehydrated in 70, 80, 95, 100% ethanol, and delipidized in 100% chloroform. The sections were pre-incubated in hybridization buffer (Sigma, St. Louis, MO, USA) and hybridized at 55°C for 24 hours with the DIG-labeled riboprobe (500 ng/ml) in the same hybridization buffer containing 50% formamide. The sections were then washed extensively in 2X, 1X, 0.1X SSC, in wash buffer and finally, in blocking buffer for 30 minutes at room temperature (DIG Wash and Block, Roche). The hybridized probes were detected using an anti-DIG alkaline phosphatase-conjugated antibody (Roche Molecular Biochemicals) diluted 1:1000, which was visualized with the alkaline phosphatase substrates nitroblue tetrazolium chloride and 5-bromo-4-chloro-3-indolyl phosphate diluted in a solution containing levamisole (Sigma). The reaction was developed for 1–2 hours at room temperature under observation to determine the optimal signal to noise ratio, and it was then quenched by rinsing in 1X SSC. The sections were subsequently air-dried, mounted in Cytoseal 60 (Electron Microscopy Sciences, Hatfield, Pensilvania, USA) and visualized on an Olympus (Tokyo, Japan) BX51 microscope.

### Mouse line

Smad3 wild-type and knockout mice were obtained by breeding heterozygous mice, and they were characterized by PCR analysis of tail biopsies [22]. 3-4-month-old female mice were group housed, maintained on a 12/12 hour light/dark cycle, and provided with *ad libitum* access to food and water. The half-lives of female Smad3^-/-^ mice is 16.2 ± 2.0 months. All procedures involving mice were carried out in accordance with EU and Spanish legislation on the care and use of experimental animals. The stage of the estrous cycle was determined for a minimum of two weeks before treatment by examining the appearance of the vagina [76], with more than 95% of animals found to be cycling normally.

### BrdU treatment

Labeling of hippocampal dividing cells was performed by administering i.p. injections of 5-bromo-2-deoxyuridine (BrdU, 10 mg/ml; Sigma) dissolved in 0.9% NaCl/7 mM NaOH. Only female mice on the first day of diestrus were used, thereby ensuring that each mouse would experience the same amount of time in each stage of the estrous cycle during the injection period [77]. To label dividing or recently divided cells in sufficient numbers for quantification, mice received once daily injections of BrdU (100 mg/Kg) at 5 p.m. on five consecutive days. For proliferative studies, mice were sacrificed 2 days after the final injection. For the differentiation analyses to follow the commitment of these newly divided cells, mice were sacrificed 28 days after the last injection. BrdU pulse-labeling assays were performed by injecting a single dose of BrdU (150 mg/Kg) and sacrificing the mice 30 minutes, 8 hours or 24 hours later.

### Immunohistochemistry

Standard chromogenic immunoperoxidase immunohistochemistry was performed [22] using mouse anti-BrdU (1:100; Dako, Copenhagen, Denmark) and rabbit anti-Ki-67 (1:500; Abcam, Cambridge, UK) primary antibodies. For the detection of BrdU-labeled cells, sections were pretreated for 30 min in 2 N HCl at 37°C to denature the DNA, and they were then incubated for 10 min in sodium borate (100 mM, pH 8.5) to neutralize any residual acid. The Vectastain peroxidase kit (Vector Labs, Burlingame, CA, USA) was used in accordance with the manufacturer’s instructions. Double immunofluorescence was performed using antigen retrieval and the following antibodies: rabbit anti-Smad3 (1:150; Abcam), rat anti-BrdU (1:1500; Abcam), rabbit anti-phospho-Smad3 (1:200; Cell Signal, Danver, MA, USA), mouse anti-nestin (1:200; Millipore, Billerica, MA, USA); mouse anti-GFAP (1:1500; Chemicon, Temecula, CA, USA), mouse anti-SOX2 (1:1000; Calbiochem, San Diego, CA, USA), mouse anti-Mash1 (1:100; BD Biosciences, San José, CA, USA), mouse anti-NeuN (1:400; Chemicon), goat anti-DCX (1:300; Santa Cruz, Dallas, TX, USA), rabbit anti-S100β (1:1000; Millipore), rabbit anti-pHisH3 (1:8000; Santa Cruz), and rabbit anti-activated caspase 3 (1:800; Cell Signal). Nissl staining of hippocampal sections was performed using 0.1% cresyl violet (Sigma).

### Cell counting and volumetric analysis

All morphological analyses were performed blind, on coded slices, using a stereological system, as described previously [22]. BrdU-ir cells were counted in every fifth section (25 μm thick sections, 125 μm apart) covering the first 2 mm of the DG from Smad3^-/-^ and Smad3^+/+^ mice. Each section was observed at low magnification (objectives 10X and 2X), and an atlas was generated with contours drawn for the GCL and the hilus, which was used as a guide. The SGZ is defined as a 2 nucleus-wide zone below the apparent border between the GCL and the hilus [78]. The hilus was defined as the area enclosed by the GCL and a virtual straight line joining the tips of its two blades [79]. The volume of the GCL and hilus was estimated using a stereological system by summing the traced area for each section, and multiplying this by the section thickness and sampling interval. Pyknotic cells were identified through their darkly stained and condensed nucleus, suggestive of chromatin condensation associated with cell death [80]. Nuclei labeled with BrdU were counted using a 40X objective, excluding those cells with a diameter less than 4 μm. The total number of BrdU-ir cells was estimated by multiplying the number of profiles by the sampling interval. For cell phenotyping, co-localization of Smad3 and BrdU with the different markers was assessed by confocal microscopy [22].

### Electrophysiological recordings

Hippocampal slices were prepared from female Smad3^+/+^ and Smad3^-/-^ mice when they reached the first day of diestrus. Mice were deeply anesthetized with isoflurane and decapitated, and their brain was removed rapidly and immersed in ice-cold standard medium containing (in mM): 119 NaCl, 2.3 KCl, 1.3 MgSO_4_, 2.5 CaCl_2_, 26.2 NaHCO_3_, 1 NaH_2_PO_4_ and 11 glucose, saturated with 95% O_2_ and 5% CO_2_ to maintain the pH at 7.4. Transverse 400 μm thick vibratome slices were obtained at 4°C (1000plus, St. Louis, MO, USA) and they were maintained in an interface holding chamber [81] at room temperature (21-25°C) and allowed to stabilize for at least 2 h. Individual slices were transferred to an open submersion-type recording chamber and perfused continuously (flow rate 1.5-2 ml/min; 30-32°C), measuring the osmolarity of the perfused solutions (Micro-osmometer 3MO, Advanced Instruments, Norwood, MA, USA).

fEPSPs were obtained by electrically stimulating the MPP with biphasic electrical pulses (60 μs) using bipolar tungsten-insulated microelectrodes (1 MΩ: S88 stimulator, Grass Instrument Co., Quincy, MA, USA). The fEPSPs were recorded from the medial molecular layer of the DG using a glass capillary microelectrode filled with standard medium (tip resistance 1–3 MΩ) and connected to a P15 amplifier (Grass Instrument Co.). A paired-pulse stimulus at a 50 ms interval was applied to confirm MPP stimulation [35]. The intensity of the stimulation used was that necessary to obtain one third of the maximum amplitude response and only slices with a maximum fEPSP amplitude greater than 1 mV were considered. After 15 min of a stable baseline response (0.05 Hz frequency stimulation), a tetanic stimulation was applied to induce LTP (four trains of 1 s each, 100 Hz within the train, repeated every 15 s). To evoke LTP in the CA1, electrodes were positioned in the stratum radiatum to stimulate the Schaffer collateral pathway. Recording microelectrodes were positioned 200–600 μm from the stimulating electrode in the same stratum.

Evoked responses were low-pass filtered at 3 kHz, digitized at 10 kHz (Digidata 1200 interface, Molecular Devices) and stored (Axotape software, Molecular Devices, Sunnyvala, CA, USA). To determine synaptic strength, the initial decay of the fEPSP slope was measured 2 ms after the stimulus (Clampfit software, Molecular Devices). The values presented for each minute in the figures are the average of three consecutive responses. The data were normalized to the averaged value of the fEPSP slope (100%) measured 15 min prior to tetanus administration (stable baseline response).

### Statistical analysis

SigmaStat software (Chicago, IL, USA) was used for all the analyses and the data were expressed as the mean ± s.e.m. The normal distribution of the data was assessed and differences between the means were analyzed with the unpaired Student’s *t*-test when investigating the effect of genotype on one variable. The effect of genotype on more than one variable was assessed using a two-way ANOVA, followed by a Holm-Sidak’ *post hoc* test. In all analyses, the null hypothesis was rejected at the 0.05 level: (*), (**), and (***) indicate P ≤ 0.05, P ≤ 0.01 and P ≤ 0.001, respectively.

## Abbreviations

AHN: Adult hippocampal neurogenesis; Cdk: Cyclin-dependent kinases; DCX: Doublecortin; DG: Dentate gyrus; fEPSPs: Evoked field excitatory postsynaptic potentials; GCL: Mature granule cell layer; GFAP: Glial fibrillary acidic protein; HFS: High frequency stimulation; -ir: -Immunoreactive; i.c.v.: intracerebroventricular; LTP: Long-term potentiation; MPP: Medial perforant path; NeuN: Neuronal specific nuclear protein; NPC: Neural precursors cells; RGL: Radial glia-like cells; SGZ: Subgranular zone; TGF-β: Transforming growth factor-β.

## Competing interests

The authors have no competing interests to declare.

## Authors’ contributions

STG and MIC carried out the molecular and cell biology experiments. MDM performed the electrophysiology, analyzed the data obtained and worked on the manuscript. ASC designed the study, carried out and supervised the experiments, analyzed the results, prepared the figures and wrote the manuscript. All authors have read and approved the final version of the manuscript.
